# Imaging analytical technique to assess gastrointestinal motility *in vivo* using zebrafish larvae with diabetes mellitus-like traits

**DOI:** 10.1371/journal.pone.0314515

**Published:** 2024-12-02

**Authors:** Jessica C. M. Hui, Peng Du, Sarah E. Webb, Julia Y. H. Liu, Man Piu Ngan, Zengbing Lu, Heidi S. H. Ng, Lingqing Yang, Aleena Khalid, Luping Liu, Zitong Li, Yingyi Deng, Dexuan Cui, John A. Rudd

**Affiliations:** 1 School of Biomedical Sciences, Faculty of Medicine, The Chinese University of Hong Kong, Hong Kong SAR, China; 2 Auckland Bioengineering Institute, University of Auckland, Auckland, New Zealand; 3 Division of Life Science, The Hong Kong University of Science and Technology, Hong Kong SAR, China; Cinvestav-IPN, MEXICO

## Abstract

In diabetes mellitus (DM), the prevalence of gastrointestinal (GI) complications, including constipation, diarrhoea, gastroparesis, and/or enteropathy, can be up to ~75%. In this study, we compared three zebrafish larvae models of DM and established an analytical protocol for GI motility. Larvae were fed with either a standard diet (SD; control), or one of three diets to induce a DM-like phenotype: excessive feeding of SD food (ED), a high-fat diet (HFD), or exposing SD-fed larvae to 30 mmol/L glucose (SDG). DM was confirmed using a body-mass index, assessment of adipose deposit areas, two glucose assays, and one insulin assay. An analytical technique, whereby GI motility was quantified using pixel differences to track displacement along the centreline of the anterior, middle, and posterior intestine (AI, MI, and PI, respectively), was developed. Our results indicated that clear DM-like traits were observed in the HFD and SGD models, but not the ED model. In the SD controls, the AI showed similar anterograde and retrograde contractions indicating normal GI mixing; the MI exhibited more prominent forward contractions, and the PI showed distinct rectal waves. Compared to the SD, the HFD and SDG models exhibited significantly increased and decreased contraction velocities and could be used as models of diarrhoea and constipation in DM, respectively, while the ED model showed comparatively little change in motility. Together, these data indicate that complex changes in GI motility are associated with diet and therapeutics used to alleviate GI complications in DM should take these into account. Ultimately, the HFD and SDG models can be used to investigate different aspects of GI motility in association to DM. Hence, zebrafish are a useful model for studying GI dysfunctions due to DM and/or DM medication side-effects.

## Introduction

According to the World Health Organisation, there are currently over 500 million people globally who have diabetes mellitus (DM), and each year 1.5 million people die of this disease [1]. One in ten adults are estimated to be diagnosed with DM, and the number to DM patients were predicted to increase 45% from 2021 to 2045 [2]. There are two main types of DM, type 1 and type 2. While the former tends to affect young people, the latter can occur at any age although it usually starts later in life due to poor diet and a sedentary lifestyle. Indeed, type 2 DM accounts for 90% of all cases [2], and there is strong evidence of a link between this disease and obesity [3]. Nevertheless, both types of DM can lead to a range of serious complications due to damage to the heart, blood vessels, eyes, kidneys and nerves, as well as affect the immune system [4, 5]. Complications involving the gastrointestinal (GI) tract are also prominent. Hauschildt *et al*. proposed that GI complications result from the gradual deterioration of gastric motility, which is proposed to be caused by elevated insulin levels and consistent hyperglycaemia [6]. This is because changes in the blood glucose induce effects on the release of hormones such as glucagon-like peptide-1 (GLP-1), motilin, ghrelin, peptide YY, gastric inhibitory peptide (GIP) and gastric inhibitory polypeptides, which modulate various gastric functions [6, 7]. The deterioration in these contractions can lead to gastroesophageal reflux disease (GERD) and gastroparesis [8]. It has recently been reported that approximately 30% of diabetic patients who suffer from gastroparesis, have an increased risk of intestinal cancer [9]. Another study found that in the GI tract of a patient with DM, the volume of the gut pacemaker interstitial cells of Cajal (ICC) was reduced, and the inhibitory innervation was decreased while the excitatory innervation and increased [10].

It can be difficult to recognise and treat the DM-based GI complications because some regularly used diabetic medications (including metformin, statins, and incretin-based treatments) might also have intestinal side-effects, such as diarrhoea, constipation, faecal inconsistence and gastric dysmotility [11]. Indeed, since 1981, the U.S. Food and Drug Administration (authorised nearly 60 anti-diabetic medications for their capacity to boost insulin production and sensitivity, and/or slow down the rate of glucose absorption from the GI tract, but treatment also affected the normal motility and functioning of the gut [12]. However, there is a gap in our understanding of the links between DM, diabetic medications, and gastric motility, partly due to the fact that the mechanisms involved in DM-induced gastric dysmotility is difficult to study. For instance, the effects of hyperglycaemia on GI motility is a ‘chicken or egg’ causality dilemma, and it is still unclear which is precedent [13]. For instance, changes in gastric emptying can influence postprandial blood glucose levels, and delayed gastric emptying attributes to poor glycaemic control due to unpredictable food transportation to the duodenum [14].

There are a number of pre-clinical animal models for investigating the effects of DM on the GI tract. Common models use rats and mice treated with streptozotocin and/or high-fat diet with direct glucose feeding has been utilized to study DM [6, 15]. In such mammalian models, GI motility studies are usually invasive, with the GI tract having to be removed and studied *ex vivo* [16]. In contrast, the transparency of zebrafish larvae provides a huge advantage for GI motility studies as peristaltic contractions can be readily visualised and analysed *in vivo*.

The zebrafish is increasingly being used in research due to its many advantages, such as easy maintenance, rapid development, and transparency in the embryonic and early larval stages. There are various zebrafish models that exhibit insulin resistance and hyperglycaemia, including the diet-induced obese and glucose immersion models [17]. Various diet-induced zebrafish models have also been developed. These include a high fat diet (HFD) comprising whipped cream and/or chicken egg yolk; over-feeding with artemia; or a combination of both diets, and both were shown to increase the triacylglycerol levels and induce hyperglycaemia in both larvae and adults [17]. Immersion in glucose solution was also shown to cause hyperglycaemia in larvae and adult zebrafish as well [18]. Despite these various DM models being developed, only a few studies have investigated the impact of diabetes in zebrafish and demonstrated that the fish exhibited certain complications such as an impaired immune system, retinopathy, neuropathy, and nephropathy [19, 20]. However, to date, very few studies have explored the intestinal complications of DM, the effect of DM medications on the GI tract, or the treatment of DM-associated GI symptoms.

In zebrafish, the GI tract becomes functional at 34 hours post fertilization (hpf), and contractions have been recorded as early as 36 hpf [21]. The onset of exogenous feeding starts at 5 days post fertilisation (dpf), and this coincides with the depletion of the yolk cell and opening of the mouth [21]. The GI tract of zebrafish can be divided into three sections (anterior, middle, and posterior) depending on the morphology as well as the type and density of cells present. The anterior intestine (AI) comprises the intestinal bulb, which lies ventral to the swim bladder and serves as a reservoir, similar to the mammalian stomach. Of the three regions, this has the largest luminal size, and it is highly folded and packed with the enteroendocrine and absorptive cells, which are used for digestion and absorption [21]. The middle region or mid-intestine (MI) is the longest region of the gut. The size of the lumen is noticeably smaller than in the intestinal bulb, but it is also folded and contains absorptive cells [21]. The posterior intestine (PI) is the shortest of the three in length, has the shortest epithelial folds, and lacks absorptive enterocytes; this is mainly for water absorption and waste expulsion [21].

Before the onset of exogenous feeding, contractile movements of the GI tract are modulated by the release of acetylcholine and nitric oxide (NO). Enteric neurons expressing neurokinin A (NKA) and pituitary adenylate cyclase-activating polypeptide (PACAP) also modulate GI motility [22]. NO is an inhibitory mediator that leads to smooth muscle relaxation [22], and several nitric oxide synthase (NOS) enzymes have been identified in zebrafish, which are responsible for its production [23]. The GI tract of zebrafish also contains ICC; these are most abundant and active in the mid-intestine where they rhythmically depolarise and initiate action potentials to generate the slow waves of contraction and communicative neurotransmission required for GI motility [24]. ICC express kit receptor tyrosine kinase as well as several transmembrane glycoproteins (e.g., CD 34 and CD 44) and so these can all be used as biomarkers of these cells [24]. In addition, a more selective marker of ICC is the Ca^2+^-activated Cl^-^ channel, anoctamin-1 (Ano1), as this has been shown to be linked to slow wave activity and pacemaker activity in these cells [24]. It has also been shown that ghrelin, leptin, cholecystokinin (CCK), orexin and neuropeptide Y (NPY) can be used as markers of zebrafish appetite and feeding behaviour [25]. Moreover, insulin-like growth factors (igf-1) are structurally similar to insulin and while they have the highest affinity to igf-1 receptors, they can also bind to insulin receptors [26]. Several studies have suggested that low IGF levels lead to insulin resistance, high blood pressure and high cholesterol, and that releasing more inflammatory markers can exacerbate diabetic complications [27].

Typical post-processing of GI motility in zebrafish included mearing the transit times of bolus over a set distance in the gut, the whole-gut transit time [28]. Image-based techniques, such as kymography has been used to quantify the spatiotemporal aspects of gut motility [28]. More advanced techniques such as image velocimetry and spectral analysis of the gut were also employed to develop quantitative spatiotemporal maps (QSTMaps), which reported the frequency, velocity, amplitude and distance, in both the longitudinal and circumference direction of the gut [16]. More pixel-based techniques, have also been adapted to quantify motility over very short segments (i.e., <400 μm) of the gut [16, 29]. Together, these techniques provide a suite of tools to extract useful kinematic information that can be further categorised to study the impact of different interventions.

The main aim of this study was to utilize DM-like zebrafish larvae models to derive quantitative metrics that can meaningfully measure GI motility patterns. Here, we utilized various previously well-established zebrafish larvae models of DM to study GI motility patterns. We hypothesized that similar peristaltic functions can be observed in zebrafish larvae as in mammals. In addition, we also hypothesized that different diets will influence the GI motility patterns in terms of contraction numbers, velocity, and frequency. This work helps to establish a zebrafish model of DM to investigate GI motility, which will be useful for investigating the effects of different DM drugs on the GI tract and determine whether DM traits observed in the larvae will persist to the adult fish.

## Materials and methods

### Zebrafish husbandry and model assembly

All zebrafish experiments were conducted under licence from the Government of HKSAR and with permission from the Animal Experimentation Ethics Committee of the Chinese University of Hong Kong (Ref No. 19-242-MIS). Wild-type AB strain zebrafish (*Danio rerio*) were obtained from the European Zebrafish Resource Center (EZRC; Karlsruhe Institute of Technology, Germany). All larvae and adults were maintained on a 14-h light/10-h dark cycle to stimulate spawning, in water at 25°C -28°C, with pH 7.4–7.6 and 300 μS—400 μS conductivity. No more than 30 adult zebrafishes were placed per 3 L tank. Female and male adults were bred in 3:2 ratio and embryos were collected and raised in fish medium comprising water purified by reverse osmosis containing 33 g/L Instant Ocean synthetic salt (Aquarium System, Inc., Mentor, OH, USA) until 5 dpf.

At 5 dpf, control larvae were fed with Gemma diet (Skretting, Utah, USA; at 20 mg—50 mg per 100 larvae); this was considered to be a so-called ‘standard-diet’ (SD). In addition, three feeding methods were implemented to initiate hyperglycaemia and/or alter the insulin levels in larvae. 1) Excess diet (ED): Larvae were overfed with Gemma diet at ~120 mg per 100 larvae. 2) High fat diet (HFD): Larvae were fed with 5% egg yolk suspension [17]. 3) Standard diet plus glucose (SDG): Larvae were fed with the SD and immersed in 30 mmol/L glucose, a change in medium each day. These various feeding regimes were conducted from 5 dpf to 7 dpf; three times a day (around 9 AM, 12PM and 5 PM) and subsequent experimental procedures were conducted accordingly, with approximately 480 larvaein total.

### Model validation

Each diabetic model was validated in the following ways. 1) A modified BMI analysis: The weight was measured (in the absence of water) with an analytical balance (KERN ADJ 200–4; Kern & Sohn, Balingen, Germany) and the length of the larvae was measured (from the head to tail) from photomicrographs. The BMI was calculated using the following formula: mg/mm^2^ [30]. In patients, several tests were conducted to diagnosis DM, blood glucose tests such as glucose at fasting state, glucose tolerance test and random blood glucose test; haemoglobin A1C test [31]. Here, we conducted 2) a fasting glucose assay; 3) a random time-points glucose assay; and 4) an insulin assay on whole-body lysates. The glucose assays were conducted using Amplex Red Glucose/Glucose Oxidase assay kits (Life Technologies Limited, Hong Kong, China) and insulin content was determined using Fish Insulin ELISA kits (Cat No. MBS269953, MyBioSource, San Diego, USA), according to the manufacturer’s instructions. The glucose- and insulin-assays were conducted immediately for the SD controls and the ED and HFD models. For the SDG model, the larvae were washed 3 times with fish medium before carrying out these assays.

To visualize the adipose deposits, live larvae were stained with fish medium containing 0.5 μg/ml BODIPY-FL C5 (Invitrogen, ThermoFisher Scientific, MA, USA) overnight at 28°C. The larvae were then washed at least 3 x 5 min with zebrafish medium, prior to imaging.

The distribution of insulin in the pancreas was determined by immunohistochemistry. Larvae were fixed with phosphate-buffered saline (PBS) containing 4% paraformaldehyde overnight at 4°C and then washed three times with PBS containing 1% Triton-X (PBTriton) for 5 min before they were incubated with a rabbit anti-insulin primary antibody (C27C9; Cell Signalling) at 1:100 dilution overnight at 4°C followed by a donkey Alexa Fluor Plus 488-tagged anti-rabbit IgG (H+L) secondary antibody (A32790; Invitrogen) at 1:500 dilution for 4 h.

### Imaging the intestinal tract for motility analysis

**Fig 1:**
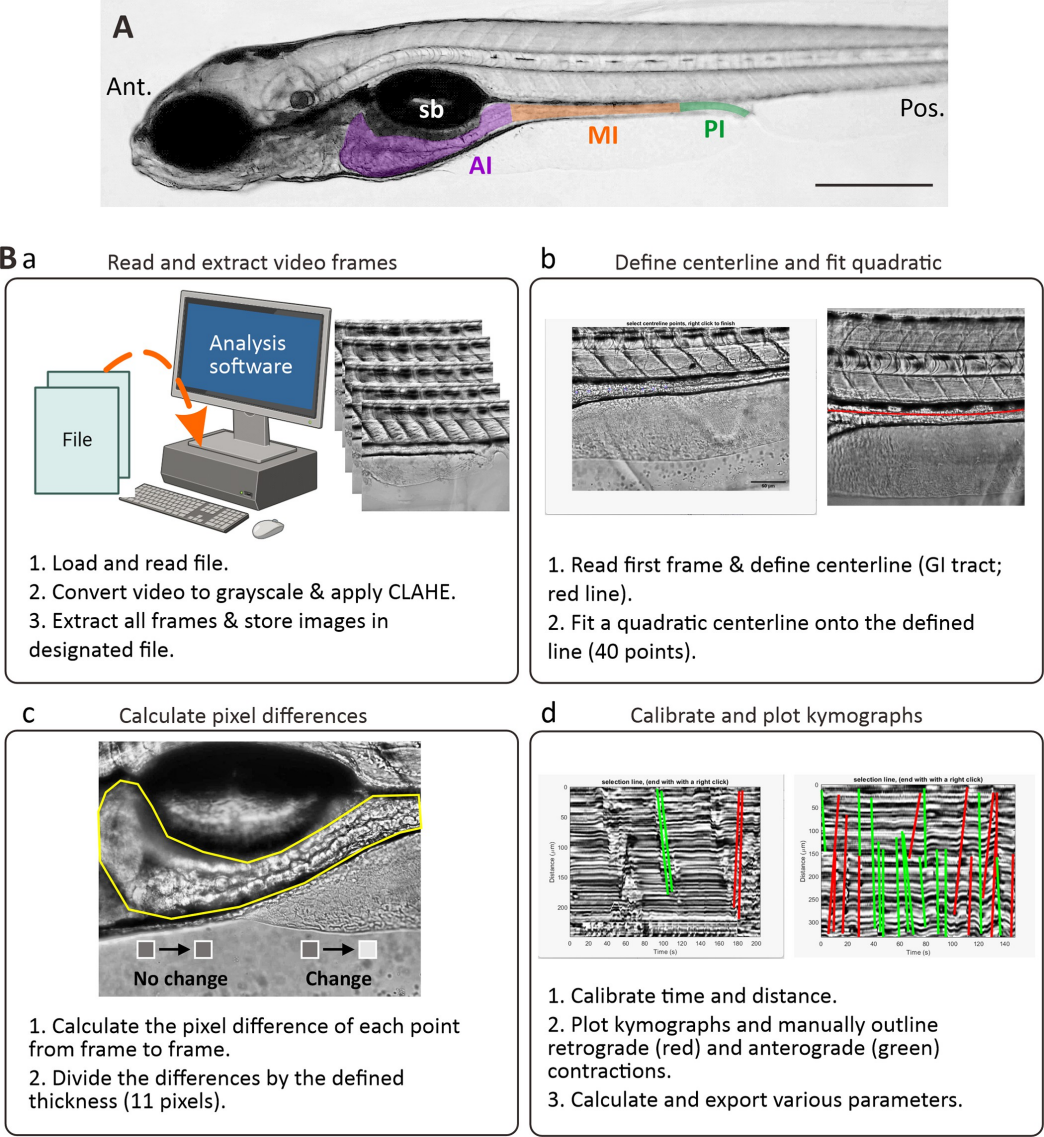
The kymography protocol. (A) Bright-field image of a lateral view of a zebrafish larva at 7 dpf showing the anterior intestine (AI, purple), mid-intestine (MI, orange) and posterior intestine (PI; green). Sb is swim bladder. Ant. And Pos. are anterior and posterior, respectively. Scale bar: 200 μm. (B) Schematic to demonstrate the four main steps (Ba-Bd) used to analyse the imaging data via kymography. In (Bd), the green and red lines are indications of contractions in the anterograde and retrograde direction, respectively. There were drawn using line tools available in MATLAB (R2018b, MathWorks, Natick, MA, USA).

Zebrafish larvae at 7 dpf were fasted for at least 2 hours and anesthetized in fish medium containing 100 μg/L MS-222 for approximately 2 min. Experiments were conducted twice a day (repeats), in the morning (9 AM) and at noon (12 PM), after the larvae were fasted for at least 2 h. The first feed started at 9 AM, therefore if the experiment started in the morning, they skip the first feed (in clean waters), while the fish for the afternoon experiments were also isolated between 11 AM and 12 PM. They were then mounted into wells containing 1% agarose gel (Bio-Rad, CA, USA) topped with 1% low-melting gel agarose (IBI Scientific, CT, USA) on a glass-bottom dish, filled with 100 μg/L MS-222 to ensure that they were kept stable with access to moisture. All motility experiments were conducted under fasting conditions to ensure that the intestine was empty prior to imaging, to avoid inconsistencies in the pixel difference data collected. Time-lapse images were then acquired using an Olympus IX83 Inverted Microscope at 5 frames per second (fps) for 300 s in three distinct regions (anterior, middle, and posterior) of the GI tract (Fig 1A). These regions were specified as follows: The anterior intestine (AI; intestinal bulb) was the region located under the swim bladder and it had the largest lumen of the three regions. The mid-intestine (MI) was identified as being the longest region, and the posterior intestine (PI) was the segment located ventral to the last three somites [22].

### Analysis of GI motility

A custom kymographic analytical method was developed by a custom algorithm in MATLAB (R2018b, MathWorks, Natick, MA, USA). The algorithm tracked the change in density along a predefined center-line of the GI tract, from which subsequent kinematic metrics were derived to quantify motility. The algorithm consisted of the following four major steps: 1) Each frame of the time-lapse video was converted to greyscale and filtered using contrast-limited adaptive histogram equalization (CLAHE) [32]; 2) A cubic polynomial interpolation was used to fit a line representing the segment of interest along the GI tract, from at least four manually annotated points from a representative frame. The algorithm then computed the intensity values at 40 equal distant data points along the line of best fit, as shown in Fig 1Bb; 3) At each data point, the change of intensity value over time was detrended and filtered using a low-pass Butterworth filter with a cut-off frequency of 2 Hz. The intensity values were then normalized to ensure variations of signals along the GI tract did not overwhelm interpretation of segments with lower amplitude signals; 3) The normalized values along the 40 data points were visualized as a kymograph, as shown in Fig 1Bd and see S1 Video, from which manual identification of propagation of contractions was conducted (red and green lines in Fig 1Bd) using line tool in MATLAB. Based on the start and end times of the waves, the algorithm would automatically color antegrade propagations in green and retrograde propagations in red; 4) Metrics such as number of contractions (n), distance of propagation along the centerline (μm), duration of each contraction (s), interval between contractions (s), and velocity (μm/s) were extracted for all defined events.

### RNA extraction, cDNA synthesis and real-time quantitative polymerase chain reaction (RT- qPCR)

Messenger RNA was extracted from larvae at 7 dpf larvae using TRIzol^TM^ reagent (Invitrogen, Thermo Fisher Scientific, MA, USA), according to the manufacturer’s instructions. The resulting RNA pellets were dissolved in 30 μl– 50 μl Rnase-free water and incubated in a water bath at 55°C– 57.5°C for 15 min. The quality and amount of RNA were then determined using a NanoDrop 2000 Spectrophotometer (Thermo Fisher Scientific, MA, USA), and sample purity was assessed at 260/280 nm. The RNA (1 μg) was then reverse transcribed into cDNA using the TaqMan^TM^ Reverse Transcription kit (Invitrogen, Thermo Fisher Scientific, MA, USA) following the manufacturer’s instructions. The expression level of each primer set (Table 1) was determined using the Applied Biosystems^TM^ Power SYBR^TM^ Green PCR Master mix (Thermo Fisher Scientific, MA, USA) in either an ABI QuantStudio^TM^ 7 (Flex) real-time PCR system (384 wells) or an ABI ViiA7 real time PCR system (96 wells). Some of the time primers were designed in-house. These included the cell surface markers, CD34 and CD44 and the ANO1 primers. These were designed using NCBI-BLAST (https://www.ncbi.nlm.nih.gov/tools/primer-blast/) and ZFIN Genomic (https://zfin.org/action/marker/search). The other primer sequences were obtained from previously published work [23, 25, 26, 33]. All the primers (Table 1.) were prepared by Tech Dragon Ltd (http://www.techdragon.com.hk/). The following thermal cycle was used: 50°C for 2 min and 95°C for 10 min, followed by 50 cycles of 95°C for 15 s and 55°C for 1 min, and then 95°C for 15 s, 60°C for 1 min and 95°C for 15 s, with the final three setting being used to hold the samples continuously until sample/data retrieval. The expression level of each gene was normalised against that of *β-actin* (internal control), after which the relative level of expression of each amplicon was calculated by the 2^-ΔΔCt^ method.

**Table 1:** List of primers used in real-time quantitative polymerase chain reaction (RT-qPCR).

Primer	Sequence
*β-actin*	F: CGAGCAGGAGATGGGAACC
	R: CAACGGAAACGCTCATTGC
**ICC-related**	
*kita*	F: GTTATCCCACTCCTCAGATCAAGT
	R: TCACAGCTACAGTCATCACAGTGT
*kitla*	F: CACAGTTGCTGCCTATTCCA
	R: GGTGAGGAGCCACCTGAGAT
*kitlb*	F: GGCTGCATTTGAACCTGTATCC
	R: GTGTCTGCACACCCTAAAGAATCC
*CD34*	F: GCATCGCATTAAAGACTACC
	R: TCCCGCATTTTAAAGCACG
*CD44*	F: CGCTCCTTCAGTTGACACC
	R: AATGTAGTGGACAAACGC
*ANO1*	F: GATGTGCACGCGGTATTCAC
	R: CTGCTCCTCTGTGCTGTTGA
**Nitric oxidase synthase**	
*nos1*	F: AAGCCATGGCCAAGAGAGTC
	R: TCACAAGTGTCTCGTGCTCC
*nos1a*	F: ACCCTGAAGAACGTGTCACC
	R: GCACAGGCTCGATCTCTTTC
*nos2a*	F: AGTATGCCACCAACGGAGGA
	R: TCGCCGATCACACTACCATC
*nos2b*	F: GGCAACCAGGTCACTCACAA
	R: CTTGATGGCACGAACTATCCTG
**Insulin-like growth factor 1 (igf)**	
*igf-1*	F: CAATGGAACAAAGTCGGA
	R: GCACAGCACCAGTGAGAG
*igfr1a*	F: AGCACTCAGGACAGGTAGCG
	R: GACAAAGGGAGGAGGGAAAT
*igfr1b*	F: ACCTACTACGTGCTCCGCT
	R: GGGTTTTGTCTCGTCCTCC
*igfbp3*	F: AAGGGGGACGTGTGAACAT
	R: GGCAGAAACAAGAATTGGG
**Feeding and appetite**	
*ghrelin*	F: GTGTCTCGAGTCTGTGAGCG
	R: CAGCTTCTCTTCTGCCCACT
*leptin*	F: TGTTGACCAGATACGCCGAG
	R: GTCCAGCGCTTTCCCATTTG
*CCK*	F: GTTCAGTCTAATGTCGGCTCC
	R: TAGTTCGGTTAGGCTGCTGC
*orexin*	F: CTACGAGATGCTGTGCCGAG
	R: GAGTGAGAATCCCGACAGCG
*NPY*	F: TGGGGACTCTCACAGAAGGG
	R: AATACTTGGCGAGCTCCTCC

The forward (F) and reverse (R) primers used to amplify the *β-actin* gene (control) and genes related to the interstitial cells of Cajal (ICC), nitric oxide synthase (nos), insulin-like growth factor (igf) and feeding and appetite in zebrafish.

### Statistical analysis

Statistical analysis was performed using two-way ANOVA followed by Dunnett/Sidak/Tukey multiple comparison tests (Prism 8, GraphPad, CA, USA) or by unpaired t-test and corrected for multiple comparisons using the Holm-Sidak method, unless otherwise stated. All data are presented as boxplots showing the minimum, maximum, sample median and first and third quartiles. Unless otherwise stated, the values are presented in mean ± SEM.

## Results

### BMI analysis, adipose deposit area, glucose and insulin levels in DM models

**Fig 2:**
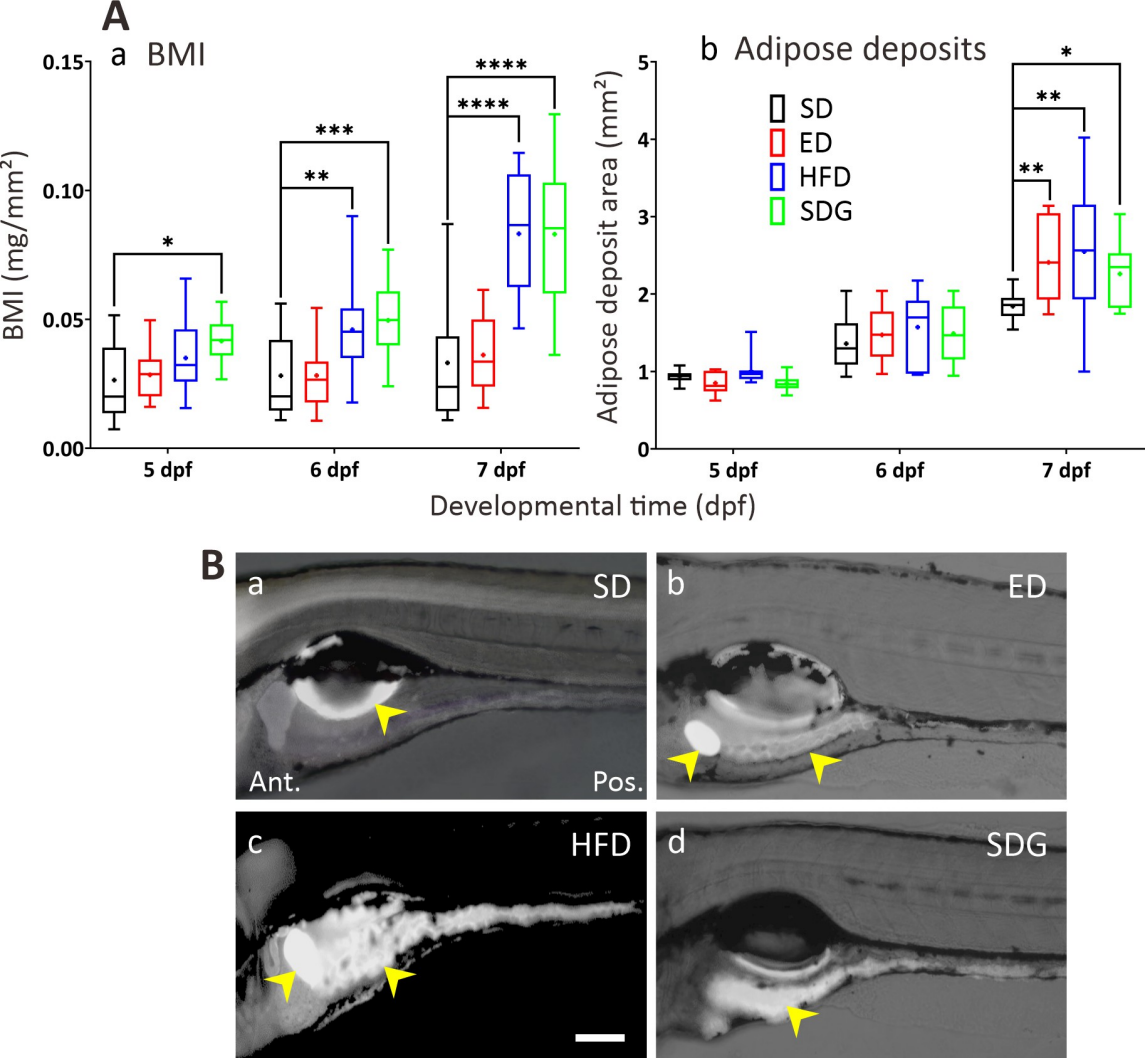
Measurements of body mass index (BMI) and adipose deposits. (A) Box plots showing the (Aa) BMI (in mg/mm^2^, n = 21) and (Ab) area of adipose deposits (in mm^2^, n = 10) measured in zebrafish larvae from 5 dpf to 7 dpf. Both plots show larvae fed a standard diet (SD, controls), or those fed an excess diet (ED), a high-fat diet (HFD), or an SD with immersion in glucose solution at 30 mmol/L (SDG). Medians are shown by horizontal lines inside the boxes, the 25^th^ and 75^th^ percentiles are shown as the bottom and tops of the boxes, and the minimum and maximum values are shown as the small horizontal lines below and above the boxes, and mean values are indicated as “+” sign. Significant differences relative to the SD controls are shown as *P<0.05, **P<0.01, ***P<0.001 and ****P<0.0001, as determined by two-way ANOVA followed by the Dunnett comparison test. (B) Representative fluorescence images of the adipose deposits (yellow arrowheads) found in (Ba) SD controls as well as in the (Bb) ED, (Bc) HFD, and (Bd) SDG models. All images were acquired when the larvae were at 7 dpf. Scale bar: 100 μm. In (Ab) and (B), the adipose deposits were visualised by incubating larvae overnight with BODIPY-FL C5 (0.5 μg/ml).

The BMI was significantly altered in the DM models compared to the SD controls (Fig 2A). The SDG model had a greater BMI than the SD controls at 5 dpf– 7 dpf, whereas the ED and HFD models only exhibited greater BMIs than the SD controls at 6 dpf and 7 dpf. The greatest increase in BMI was observed at 7 dpf in both the HFD and SDG models compared with the controls (SD: 0.033 ± 0.005 mg/mm^2^, HFD: 0.083 ± 0.005 mg/mm^2^ and SDG: 0.083 ± 0.006 mg/mm^2^; p>0.0001). A series of representative images showing adipose staining in the abdominal area of larvae at 7 dpf are shown in Fig 2B. All three DM models also showed significantly greater adipose deposit areas than the SD controls at 7 dpf (SD: 1.84 ± 0.059 mm^2^, ED: 2.41 ± 0.17 mm^2^, HFD: 2.55 ± 0.278 mm^2^, and SDG: 2.26 ± 0.14 mm^2^;p>0.05 and p<0.01), but not at 5 and 6 dpf (Fig 2Ab).

**Fig 3:**
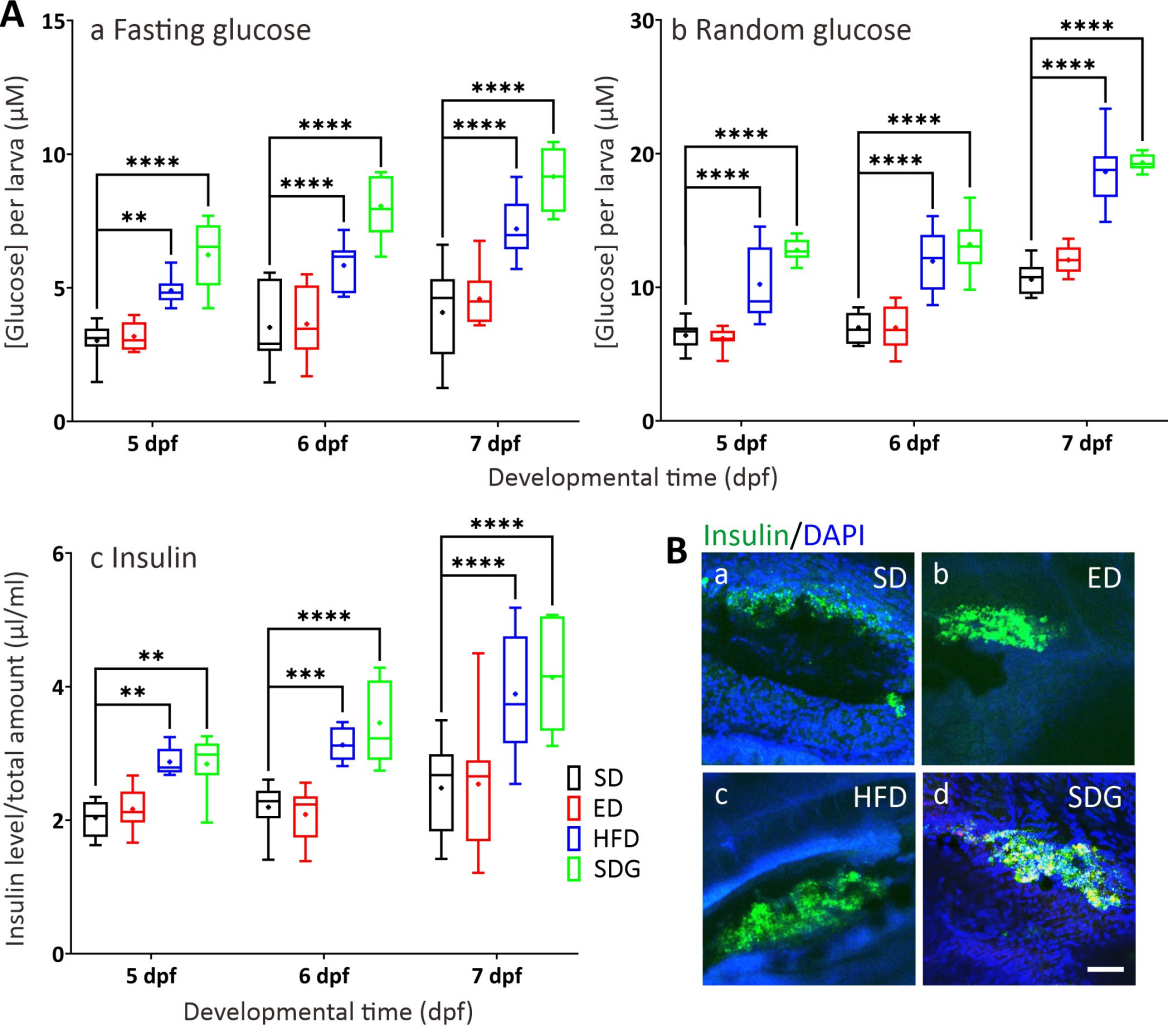
Quantification of fasting glucose, random glucose, and insulin levels. (A) Box plots showing (Aa,b) the amount of glucose (in μM) following the (Aa) fasting or (Ab) random glucose assay, and (Ac) the amount of insulin (in μl/ml), in larvae fed with the standard controls (SD), excess diet (ED), high fat diet (HFD) or SD + glucose diet (SDG), at 5 dpf, 6 dpf or 7 dpf. All the assays were repeated 9 times with n = 10 larvae each time. Medians are shown by horizontal lines inside the boxes, the 25^th^ and 75^th^ percentiles are shown as the bottom and tops of the boxes, and the minimum and maximum values are shown as the small horizontal lines below and above the boxes, and mean values are indicated as “+” sign. Significant differences relative to the SD controls are shown as *P<0.05, **P<0.01, ***P<0.001 and ****P<0.0001, as determined by two-way ANOVA followed by the Dunnett comparison test. (B) Representative fluorescence images of the localization of insulin in the pancreas of (Ba) SD, (Bb) ED, (Bc) HFD, and (Bd) SDG larvae, which were fixed at 7 dpf and immunolabeled with an anti-insulin antibody. Scale bar: 50 μm.

The fasting/ random glucose tests (Fig 3Aa and 3Ab) and insulin test (Fig 3Ac) showed that the HFD and SDG models, but not the ED model, had significantly increased levels of glucose and insulin, respectively, compared with the SD controls, at all-time points. In the fasting glucose assay (Fig 3Aa), the glucose levels in the HFD and SDG models were approximately 2 and 3 times greater, respectively, than those in the SD controls at 5 dpf. In addition, at 6 dpf and 7 dpf, both of these models exhibited an almost 2-fold increase in glucose when compared with the SD controls (6 dpf—SD: 3.52 ± 0.50 μM, HFD: 5.84 ± 0.29 μM, SDG: 8.06 ± 0.39 μM; 7 dpf—SD: 4.08 ± 0.58 μM, HFD: 7.21 ± 0.36 μM, SDG: 9.16 ± 0.38 μM). In the random glucose assay (Fig 3Ab), the glucose levels were significantly higher in the HFD and SDG models than the SD controls at 5 dpf to 7 dpf. At 7 dpf, the glucose levels were the highest in these models compared to the SD controls (SD: 10.59 ± 0.40 μM, HFD: 18.65 ± 0.82 μM, SDG: 19.33 ± 0.20 μM).

Analysis of insulin levels (Fig 3Ac) showed that insulin in the ED model were similar to the SD controls at all time points, whereas both the HFD and SDG models showed an increase of approximately 1.4 times that of the SD controls at 5 dpf and 6 dpf. At 7 dpf, the insulin levels in both these models showed an increase approximately 1.6 times higher than that of the SD controls (SD: 2.48 ± 0.23 μl/ml, HFD: 3.89 ± 0.31 μl/ml and SDG: 4.14 ± 0.27 μl/ml). Representative images of insulin immunolabeling in the pancreas of the three models and the controls at 7 dpf are shown in Fig 3B. These images showed that the HFD and SDG models both exhibited larger regions of insulin labelling when compared with the SD controls.

### Baseline GI motility analysis in the SD model

**Fig 4:**
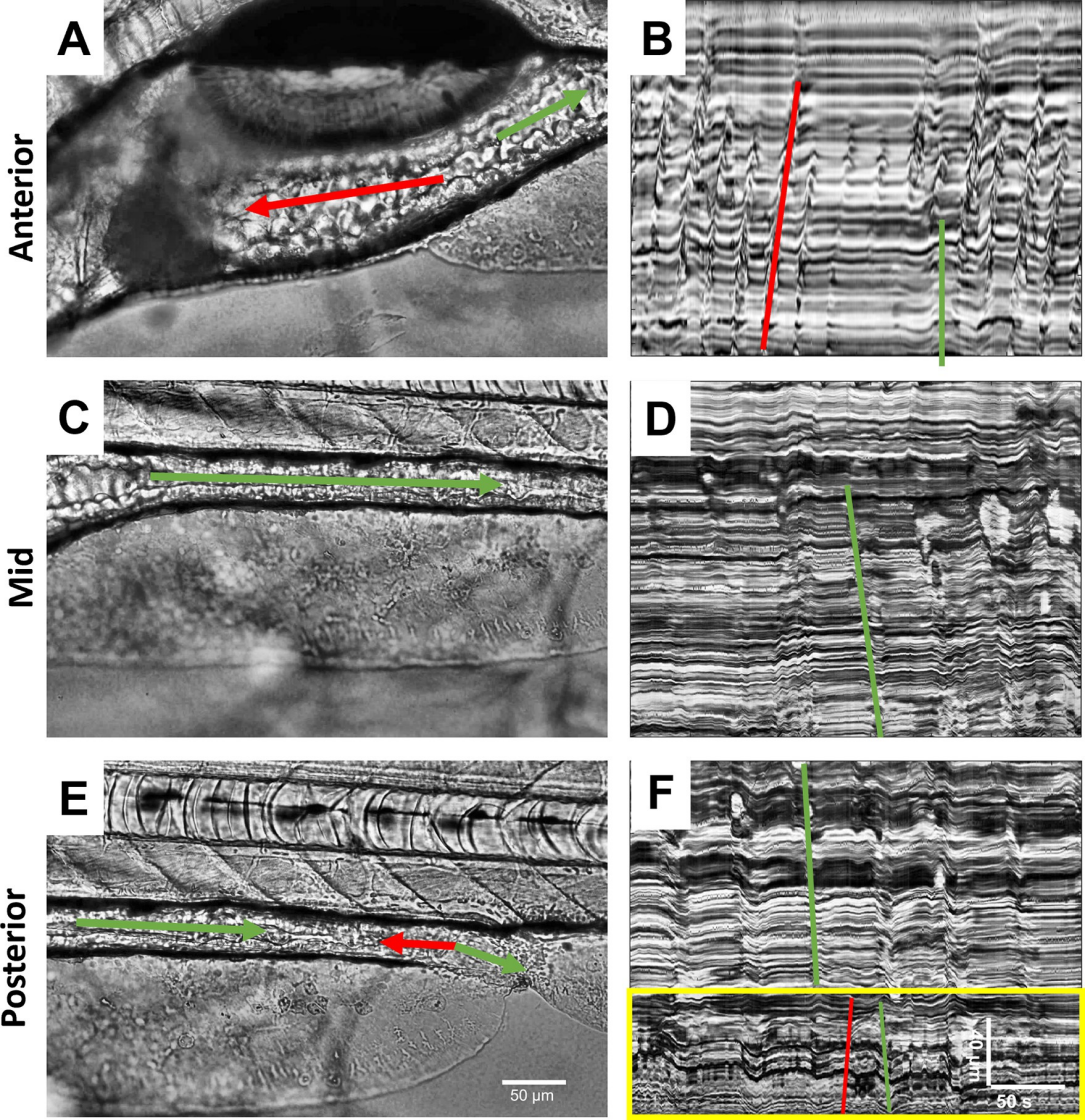
Measuring gut motility using kymography. (A,C,E) Representative bright-field images showing the (A) anterior, (C) mid and (E) posterior regions of the intestine of SD controls larvae at 7 dpf, and (B,D,F) their respective kymographs. Green and red arrows (in A, C, and E) and lines (in B, D, and F) indicate anterograde and retrograde contractile activity, respectively, and the yellow rectangle (in F) indicates the rectal waves. Scale bars are 50 μm (A,C,E) and 40 μm over 50 s (B,D,F).

A presentative sample of the AI, MI, and PI regions of the GI tract and their associated activities are shown in Fig 4. In this instance, the AI exhibited predominantly retrograde propagations, with sporadic episodes of anterograde propagations that covered similar distance as the retrograde propagation (Fig 4A). In the MI, only anterograde propagations occurred (Fig 4B). In the PI, while the top 2/3 proximal distance of the segment exhibited only anterograde propagations, a mixture of anterograde and retrograde propagations occurred in the rectal end of the GI tract (Fig 4C, bounded by yellow rectangle).

**Fig 5:**
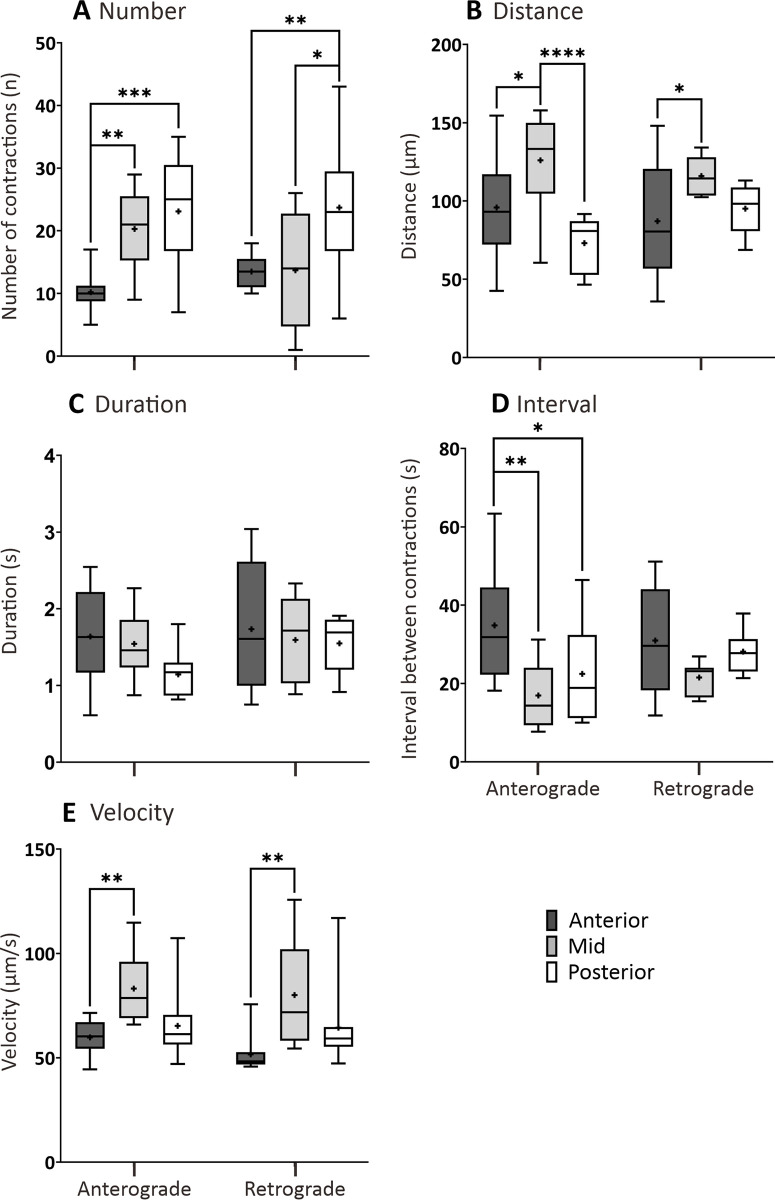
Box plots to show various parameters of GI motility determined from AB larvae at 7 dpf that were fed a standard diet (SD). The following parameters were determined by kymography analysis: (A) number, (B) distance (μm), and (C) duration (s) of contractions, (D) interval between contractions (s), and (E) contraction velocity (μm/s), in both the anterograde and retrograde direction. Medians are shown by horizontal lines inside the boxes, the 25^th^ and 75^th^ percentiles are shown as the bottom and tops of the boxes, and the minimum and maximum values are shown as the small horizontal lines below and above the boxes, and mean values are indicated as “+” sign. Significant differences were determined by two-way ANOVA followed by the Tukey/Sidak multiple comparison test and are presented as *P<0.05, **P<0.01, ***P<0.001 and ****P<0.0001. N = 10.

In SD controls larvae at 7 dpf, a comparison was made between AI, MI, and PI with regards to the number, distance, and duration of contractions as well as the interval between contractions and velocity of contractions (Fig 5). In addition, there were no significant differences between the anterograde and retrograde contractions in any of the motility GI parameters investigated. Significant inter-regional variations of contractile activities were present in the SD controls at 7 dpf (Fig 5). There were significantly more anterograde contractions in the MI and PI than in the AI, and there were significantly more retrograde contractions in the PI than in the other two regions (Fig 5A). The anterograde contractions in the MI covered the longest distance compared to the AI and PI, whereas in the retrograde direction in the MI was only significant compared to the AI (Fig 5B). No significant differences were found in the duration of propagation between the three regions (Fig 5C). The lower anterograde contraction numbers in the AI are correlated to the longer time intervals between contractions and they result in significantly greater intervals than those in the MI and PI. However, no significant differences in the intervals were found in the retrograde contractions when comparing the three intestinal regions (Fig 5D). Finally, the anterograde and retrograde contractions both exhibited highest velocity in the MI than those in the AI, but not in the PI (Fig 5E).

**Fig 6:**
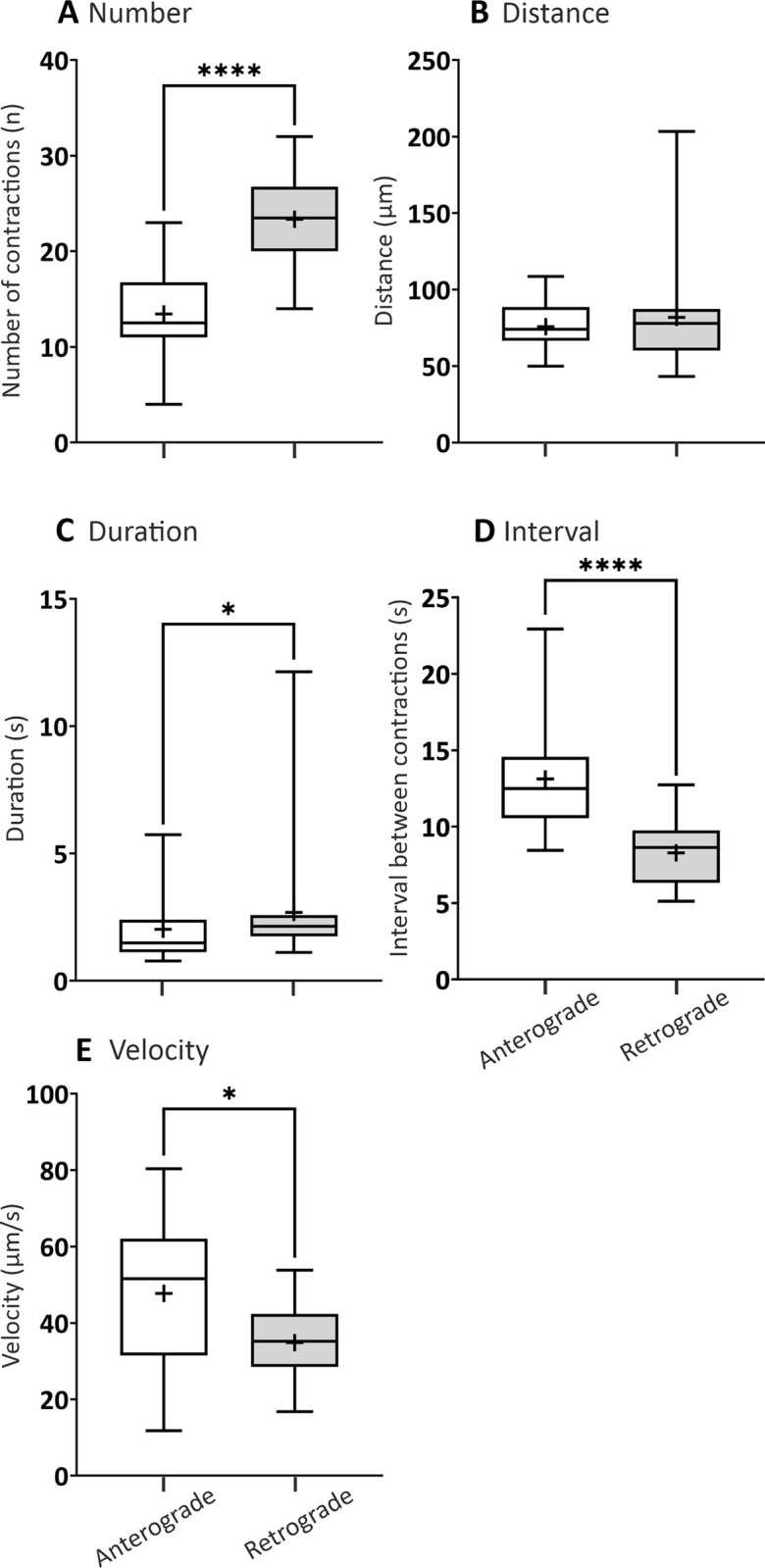
Box plots to show various parameters of the rectal waves generated in AB larvae at 7 dpf that were fed a standard diet (SD). The following parameters were determined by kymography analysis: (A) number, (B) distance (μm) and (C) duration (s) of contractions, (D) interval between contractions (s), and (E) contraction velocity (μm/s). These parameters were determined in both the anterograde and retrograde direction. Medians are shown by horizontal lines inside the boxes, the 25^th^ and 75^th^ percentiles are shown as the bottom and tops of the boxes, and the minimum and maximum values are shown as the small horizontal lines below and above the boxes, and mean values are indicated as “+” sign. Significant differences between the anterograde and retrograde actions were determined by the unpaired nonparametric Mann Whitney U test and are presented as *P<0.05 and ****P<0.0001. N = 20.

Significant differences were observed in terms of number of contractions, durations of contractions, intervals, and velocities between anterograde and retrograde propagations in the rectal waves (Fig 6). There were significantly more contractions generated in the retrograde direction than the anterograde direction (Fig 6A). There was no significant difference in the distance of the contractions (Fig 6B), but the durations were greater in the retrograde direction than anterograde direction (Fig 6C). While, the intervals between contractions and velocity of contractions were both significantly greater in the anterograde direction than in the retrograde direction (Fig 6D and 6E).

### GI motility in the ED, HFD and SDG models compared to the SD controls

**Fig 7:**
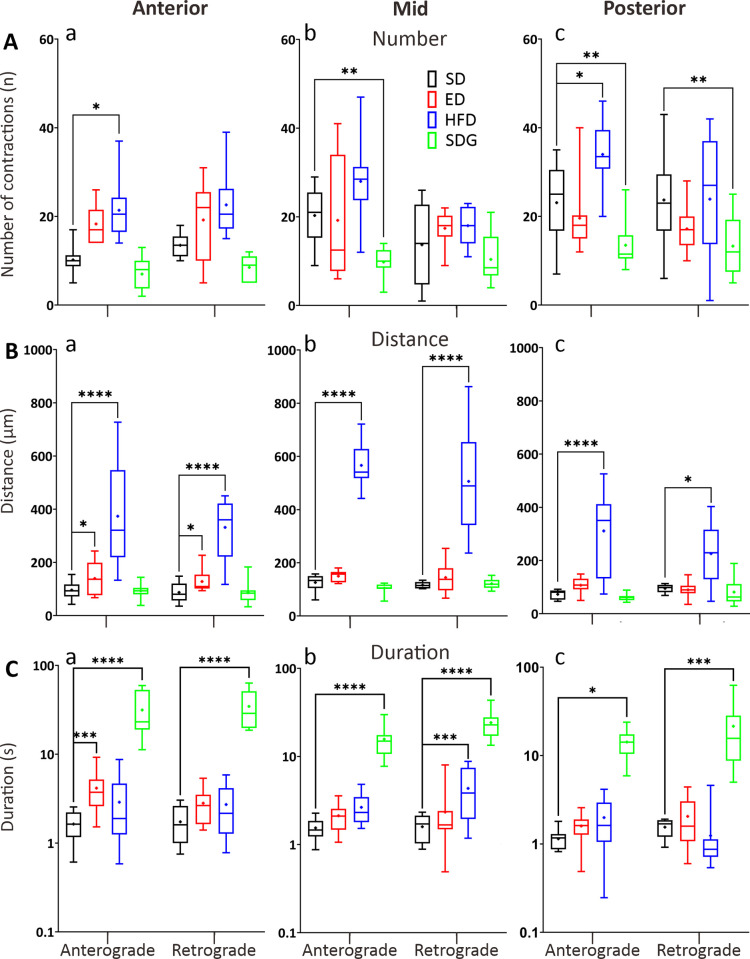
Box plots to show various parameters of GI motility in the anterior, mid, and posterior intestine of ED, HFD and SDG larvae compared with the SD controls, at 7 dpf. The following parameters were determined by kymography analysis: (A) number, (B) distance (μm), and (C) duration (s) of contractions, in both the anterograde and retrograde direction. Medians are shown by horizontal lines inside the boxes, the 25^th^ and 75^th^ percentiles are shown as the bottom and tops of the boxes, and the minimum and maximum values are shown as the small horizontal lines below and above the boxes, and mean values are indicated as “+” sign. Significant differences were determined by two-way ANOVA followed by the Sidak multiple comparison test and are presented as *P<0.05, **P<0.01, ***P<0.001 and ****P<0.0001. N = 10.

**Fig 8:**
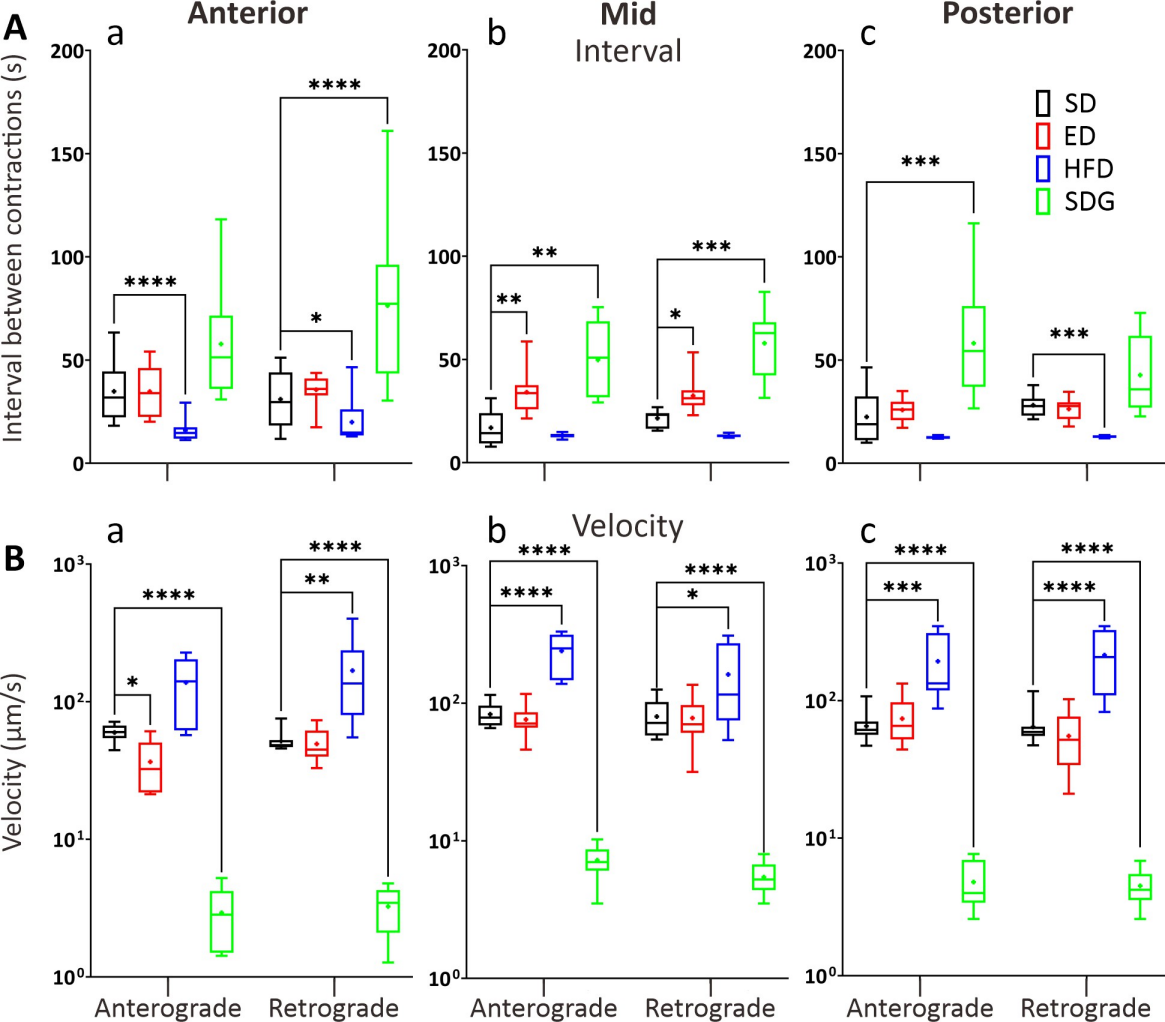
Box plots to show various parameters of GI motility in the anterior, mid, and posterior intestine of ED, HFD and SDG larvae compared with the SD controls, at 7 dpf. The (A) interval (s) and (B) velocity (μm/s) of contractions were determined in both the anterograde and retrograde direction by kymography analysis. Medians are shown by horizontal lines inside the boxes, the 25^th^ and 75^th^ percentiles are shown as the bottom and tops of the boxes, and the minimum and maximum values are shown as the small horizontal lines below and above the boxes, and mean values are indicated as “+” sign. Significant differences were determined by two-way ANOVA followed by the Sidak multiple comparison test and are presented as *P<0.05, **P<0.01, ***P<0.001 and ****P<0.0001. N = 10.

All three DM models showed changes in GI motility compared to the SD controls at 7 dpf (Figs 7 and 8). In general, only the HFD and SDG models showed changes in number of contractions (Fig 7A). The HFD model showed 35% and 19% increases in the number of anterograde contractions in the AI (21.4 ± 2.09) and PI (34.00 ± 2.42) compared to the SD controls, respectively (Fig 7Aa and 7Ac). No difference in retrograde propagation was observed in the HFD model. On the other hand, the SDG model showed a 52% increase and a 42% decrease in the number of anterograde propagations in the MI (9.8 ± 1.03) and PI (13.5 ± 1.67) compared to the SD controls, respectively (Fig 7Ab and 7Ac). In addition, the SDG model was the only model that showed 44% decrease in the number of retrograde propagations in the PI (13.3 ± 2.16) compared to the SD controls.

The ED and HFD models showed significance increases in the distance of propagation when compared with the SD controls (Fig 7B). The ED model showed a 46% and 47% increase in the distance of the anterograde (139 ± 20 μm) and retrograde (128 ± 13 μm) contractions in the AI compared to the SD controls, respectively (Fig 7Ba). No significant differences in distance of contractions were observed in other GI regions of the ED models when compared to controls. In HFD models, the distance of contractions were significantly increased in all three GI regions. The distance of the anterograde (373 ± 59 μm) and retrograde (331 ± 35 μm) contractions was increased by 59% and 58% in the AI when compared with the SD controls. In the MI (Fig 7Bb), the distance of the anterograde (566 ± 25 μm) and retrograde (506 ± 62 μm) contractions were significantly longer, by 64% and 63%, respectively in the HFD model than the SD controls. Similarly, in the PI (Fig 7Bc), the distance of the anterograde (311 ± 48 μm) and retrograde (226 ± 38 μm) contractions were significantly longer by 62% and 41%, respectively, in the HFD model than the SD controls. No significant differences in terms of contractile distances were observed between the SDG models and SD controls.

All three DM models showed significant differences in the duration of contractions when compared to the SD controls, whereas only the SDG models were significantly different from the SD controls in all three GI regions (Fig 7C). The ED model only showed significant increase in durations of anterograde contractions in the AI (Fig 7Ca), from 4.16 ± 0.72 s in the ED models to 1.64 ± 0.20 s in SD controls. In the HFD model, the duration of retrograde contractions in the MI (Fig 7Cb) were 4.35 ± 0.88 s and was significantly increased when compared to controls. In the SDG model, the duration of contractions was significantly increased in all three GI regions. The duration of the anterograde (31.49 ± 5.74 s) and retrograde (34.54 ± 5.46 s) contractions was increased by 18.2 and 18.9 times in the AI when compared with the SD controls, respectively. In the MI, the duration of the anterograde (15.72 ± 1.98 s) and retrograde (24.21 ± 2.98 s) contractions were significantly longer, by 9.2 and 14.2 times, respectively in the SDG model than the SD controls. Similarly, in the PI (Fig 7Cc), the duration of the anterograde (14.27 ± 1.65 s) and retrograde (21.48 ± 6.01 s) contractions were significantly longer by 11.5 and 12.9 times, respectively, in the SDG model than the SD controls.

The ED and SDG models showed significant increases in the intervals, whereas the HFD model showed significant decrease in intervals, compared to the SD controls (Fig 8A). The ED model showed significant increases in the MI by 101% and 51% in intervals between contractions in the anterograde and retrograde direction when compared to SD controls, respectively (Fig 8Ab); while no significant differences were observed in other GI regions. In HFD models, the intervals between retrograde contractions were significantly decreased in the AI and PI by 22% and 37% when compared with SD controls, respectively (Fig 8Aa and 8Ac). In addition, the HFD model was the only model that showed 37% decrease in the intervals between anterograde propagations in the AI compared to the SD controls. In SDG models, the intervals between anterograde and retrograde contractions were significantly increased by 190% and 170% in the MI when compared to SD controls, respectively. In addition, the intervals between contraction of SDG models in retrograde and anterograde direction were significantly increased by 42% and 44% in the AI and PI respectively when compared to SD controls.

Finally, when comparing the contraction wave velocities (Fig 8B), main differences were observed in the HFD and SDG models, whereas the ED model only showed a significant decrease in the anterograde velocity in the AI (Fig 8Ba), when compared with the SD controls (i.e., the anterograde velocity decreased by 39% in the ED model). In the HFD model, the retrograde velocities were significantly faster (by 53%) in AI when compared with the controls. In the MI of HFD model (Fig 8Bb), the anterograde and retrograde contraction velocities were significantly increased by 49 and 32% when compared with SD controls, respectively. Similarly, significantly faster velocities were recorded in the PI region of HFD models (Fig 8Bc), the velocities were increased by 49% and 53% in the anterograde and retrograde direction when compared to SD controls, respectively. In SDG models, significant slower velocities were recorded in all GI regions when compared to SD controls. In the AI, the anterograde and retrograde velocities were significantly reduced by 91% and 88%, respectively in SDG the model. In the MI, the anterograde and retrograde contraction velocities were significantly decreased by 84% and 87% when compared with SD controls. In the PI of SDG models, the velocities were decreased by 86% and 86% in the anterograde and retrograde direction when compared with SD controls, respectively.

**Fig 9:**
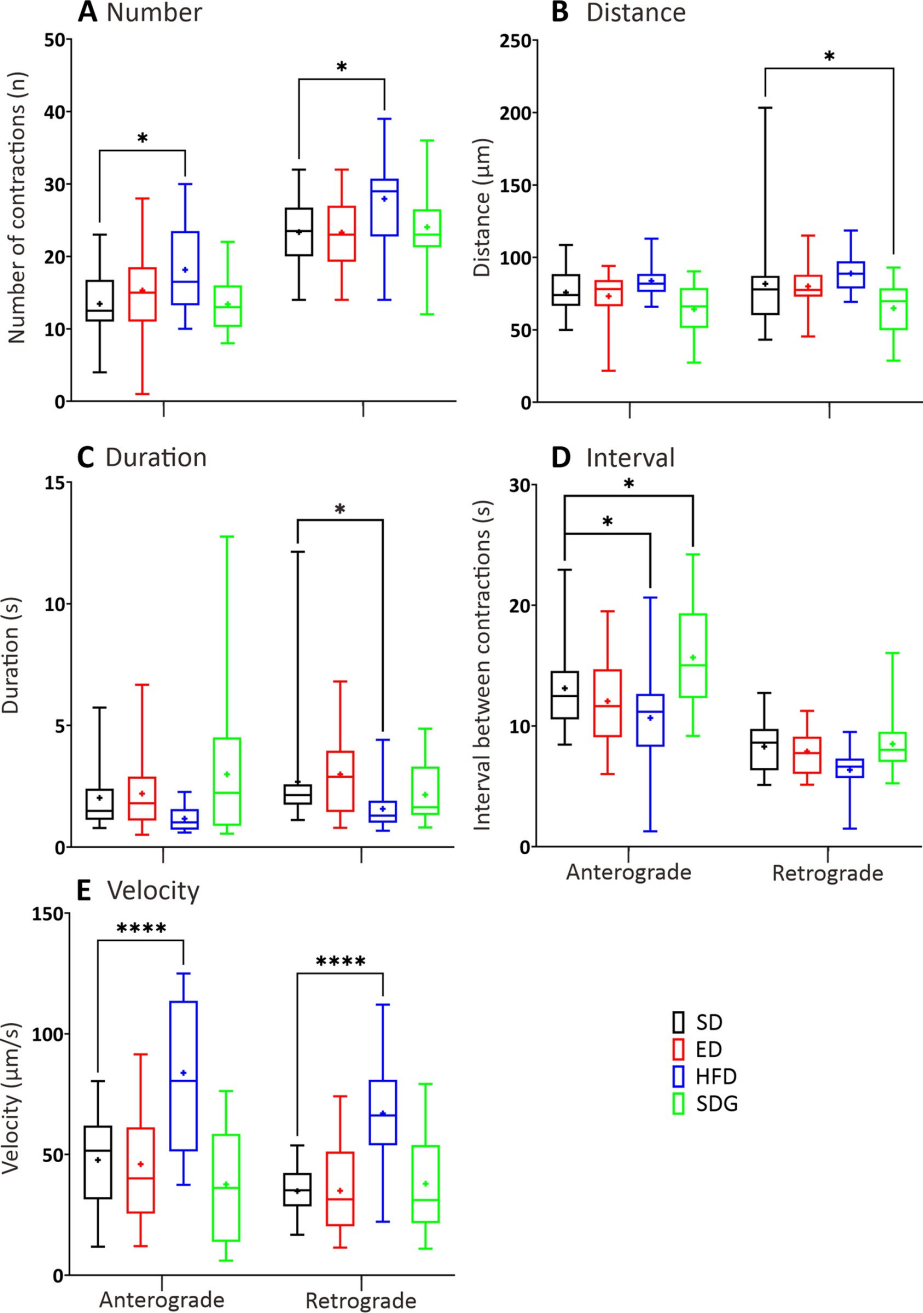
Box plots to show various parameters of the rectal waves ED, HFD and SDG larvae compared with the SD controls, at 7 dpf. The (A) number, (B) distance (μm), and (C) duration (s) of contractions, (D) interval between contractions(s), and (E) contraction velocity (μm/s) were determined in both the anterograde and retrograde direction by kymography analysis. Medians are shown by horizontal lines inside the boxes, the 25^th^ and 75^th^ percentiles are shown as the bottom and tops of the boxes, and the minimum and maximum values are shown as the small horizontal lines below and above the boxes, and mean values are indicated as “+” sign. Significant differences were determined by two-way ANOVA followed by the Sidak multiple comparison test and are presented as *P<0.05, **P<0.01, ***P<0.001 and ****P<0.0001. N = 20.

The rectal region generally showed less variation between the DM models and SD controls (Fig 9). In ED models, there were no significant differences in all GI motility metrics when compared to the rectal waves of SD controls. Only the HFD model showed significantly increase in the number of contractions in both directions, (by 15% and 9%, respectively), compared to the SD controls (Fig 9A). In the HFD model, the duration of retrograde contractions and intervals between anterograde contractions were significantly decreased by 41% and 19% respectively (Fig 9C and 9D). Finally, when comparing the rectal wave velocities between the three DM models and the SD controls, only the HFD model exhibited significant increases, by 27.47% and 31.59% in the anterograde and retrograde direction, respectively (Fig 9E). While only the SDG model showed a significant decrease of 12% in the distance of the retrograde contractions (Fig 9B). In addition, the intervals between the anterograde contractions were significantly increased in SDG models (i.e., the intervals increased by 19% in in the SDG model).

### Genetic expression levels in DM models when compared to SD larvae

**Fig 10:**
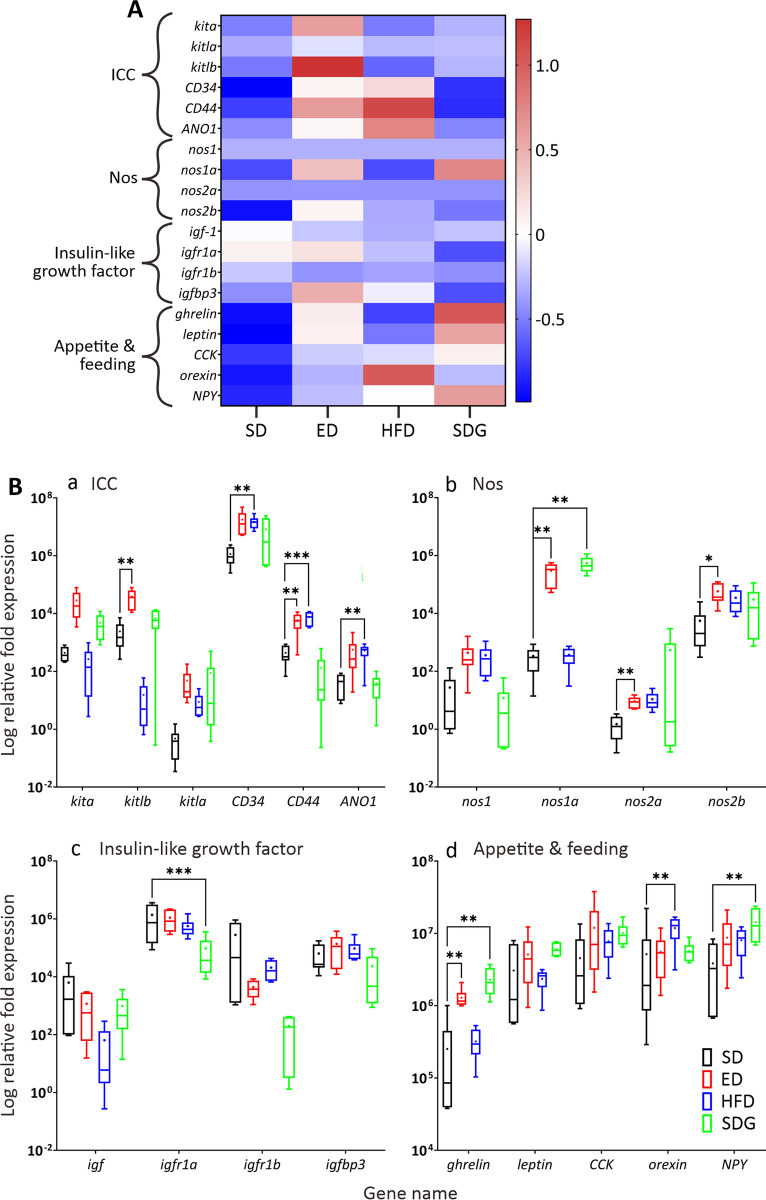
Graphs showing the qPCR data from SD, ED, HFD and SDG larvae at 7 dpf (n = 6 for each). The in heatmap (A) and boxplots (B) data for each gene were normalised and expressed as the relative fold expression compared to *β-actin*. The expression of genes related to interstitial cells of Cajal (ICC), nitric oxide synthase (Nos), insulin-like growth factor (igf), and appetite and feeding, are shown. In (A), the gene names are shown in italics on the left and the relative expression is indicated using a graded colour-scale, where each gene is assigned a z-score depending on the respective amount of down- (shades of green) or up- (shades of red) regulation between the samples. In (B), medians are shown by horizontal lines inside the boxes, the 25^th^ and 75^th^ percentiles are shown as the bottom and tops of the boxes, and the minimum and maximum values are shown as the small horizontal lines below and above the boxes, and mean values are indicated as “+” sign. Significant differences between the ED, HFD and SDG data and the SD controls were determined using the unpaired t-test (and corrected for multiple comparisons using the Holm-Sidak method), and they are shown as *P<0.05, **P<0.01, and ***P<0.001. N = 6.

Several gene targets that have been previously identified, which might impact GI motility, appetite, or the well-being of larvae, were investigated in the DM models. Several changes in gene expression levels were observed. These are shown in the heatmap (Fig 10A) and the levels of relative fold expression were plotted using boxplots (Fig 10B). The data show that several genes related to ICC were upregulated in the ED and HFD models, whereas genes related to appetite and feeding behaviour were upregulated in the HFD and SDG models. With regards to ICC-related genes, the expression levels of the ligands of the c-Kit receptor tyrosine kinase, *kitlb* and the kit marker, *CD44* were significantly upregulated in the ED model, whereas *CD34*, *CD44*, and the chloride channel–*ANO1* were all significantly upregulated in the HFD model. No significant differences in ICC markers were observed in the SDG model (Fig 10Ba). With regards to Nos-related genes (Fig 10Bb), the relative fold expression of *nos1a*, *nos2a* and *nos2b* were significantly higher in the ED model than the controls, and *nos1a* was upregulated in the SDG model; however, the HFD model did not exhibit any significant differences in the expression of the *nos* genes. With regards to the genes involved insulin-like growth factor (Fig 10Bc), the relative fold expression of *igfr1a* was significantly reduced in the SDG model, but no significant differences were observed in the ED and HFD models. Finally, the expression levels of genes associated with appetite and feeding behaviour were investigated (Fig 10Bd). The data showed that *ghrelin* was significantly upregulated in the ED and SDG models and NPY was significantly upregulated in the SDG model, when compared with SD controls. In addition, *orexin* was significantly upregulated in the HFD model.

## Discussion

In this study, various obesity- and/or diabetic-like traits were induced in zebrafish larvae by the following nutritional means: excessive diet (ED; ~6 times the standard diet; SD, which was 20 mg Gemma feed), a high-fat diet (HFD), and incubation in glucose solution (SDG) [17, 34]. The obesity and diabetic traits were confirmed by measuring the BMI and amount of adipose, as well as via two glucose assays (fasting and random) and one insulin assay. The HFD and SDG models both showed increased BMI and adipose deposits as well as elevated glucose and insulin levels. These data indicate that glucose was elevated by these different diets, and the insulin levels increased concomitantly to compensate, but were ineffective in lowering the glucose levels in the body [35]. In contrast, the ED model did not show an increase in BMI and only showed increased adipose deposits (in the abdominal area) at 7 dpf. The glucose and insulin levels were unchanged in this model compared to the SD controls. We suggest that it might represent a model of obesity, although the clinical definition obesity is mainly categorized using the BMI, which is absent in this case. As all the models exhibited prominent characteristics at 7 dpf, this time of development was employed for the subsequent GI motility analyses.

Larvae at 7 dpf were fed an SD and fasted, they are then used to define a baseline for the GI motility analysis and for comparison purposes in the different models. The SD controls showed significant inter-regional differences in terms of the motility-metrics defined (i.e., number of waves, distance of propagation, duration, interval, velocity), in the AI, MI, and PI. No differences in the metrics between antegrade and retrograde directions were seen, except in the rectal segment. Although Fujii *et al*. (2020) previously reported there to be a higher number of retrograde activities in the AI region, which appeared to attenuate in 10 dpf larvae once oral intake of food had started [36]. Instead of tracking the changes in the transverse axis as an indicator of retrograde propagation, the present studies tracked in the organal-axis of the GI tract and therefore may represent a smaller subset of retrograde activities analysed by Fujii *et* al. In future, this discrepancy may be reconciled through machine-learning approaches to automatically extract the waves without relying on manual marking.

Notably, in the present study the absence of significant differences in the intervals between retrograde contractions might be indicative of activities occurring in bursts that are independent of each other. It is likely that the bursts of GI activity could be due to the fasted state, and the presence of migrating motor complexes (MMC) phase III [37]. However, further investigation on the electrical activity of the GI tract would be required to determine their presence. Nevertheless, the absence of significant differences between the anterograde and retrograde contractions, and the lower frequency and increased intervals in the anterograde contractions in the AI were indicative of increased mixing in this region, which prevented the GI tract contents moving forward. Mixing involves both and anterograde and retrograde actions that break the bolus into smaller sizes before it enters the subsequent regions of the GI [22, 38]. After the mixing actions in the AI, the MI exhibited a dominant anterograde action, which showed increased forward contraction. The fact that expansive and frequent anterograde contractions are dominant in the MI of larvae, suggests that this region is mainly involved in the transport of food towards the posterior end for expulsion after food absorption [16, 38]. In the kymographs of the PI, long streaks of propagation waves were observed in addition to rectal waves. The propagation waves in the posterior region might be the main force that propels the GI contents for excretion or compaction [16, 29]. At the posterior end, a series of rectal wave movements were also observed. It should be noted that these were isolated from the PI contraction waves by manual means (identified at the bottom of kymographs) and using a threshold where the distance of contractions was ≤200 μm. Such waves have previously been described as short and frequent waves in both the anterograde and retrograde directions, which provide greater contractile movements and mixing motion, and thus they limit rectal filling and assist excretion [22]. More recently, it was suggested that in humans, dominant retrograde contractions at the proximal end might be due to a rectosigmoid brake, which helps to retain food and limit rectal filling [39]. In our study, the presence of rectal waves in zebrafish larvae indicates that they might assist in food/waste expulsion, however, GI tract emptying was not studied here and so it will be explored in future studies to fully understand the action of excretion.

Although there were some differences in terms of contractions between the various DM models and the SD controls at 7 dpf, all the models shared similar motility patterns. Indeed, in all cases, the lowest velocity and highest mixing action was found in the AI, whereas the greatest velocity was found in the MI, and rectal waves were observed at the PI to a greater or lesser extent depending on the model.

In the ED model, the anterior intestine exhibited contractions with increased duration and mixing when compared with the SD controls, which indicated deteriorated mixing actions of the gut in this model [29]. In addition, in the MI, longer intervals between contractions were observed, which might indicate that fewer and slower contractile actions were taking place. Such reduced contractile activities might allow more time for the absorption of food as the mid-intestine is primarily responsible for transporting the boluses of food [29]. However, the absence of significant differences in the rectal waves between the ED and SD larvae, indicates that there were no changes in GI tract emptying.

In the HFD model, GI motility was accelerated due to the increased contraction numbers and velocity and shorter intervals when compared with the SD controls. In addition, higher anterograde contraction numbers in the MI and PI might indicate more forward propagation. There was also a faster forward velocity in the rectal waves of HFD larvae, which suggests that GI tract emptying was accelerated. This contradicts previous reports, which suggest that gastric emptying is delayed after the ingestion of fat in the diet, mainly due to disruptions in the ability to sense nutrients in enteroendocrine cells [40, 41]. However, more recent studies describe increased gastric emptying with a fat-based diet [42]. This might be due to the adaptation or altered sensitivity to the hormones responsible for gastric motility [43]. In addition, several studies showed accelerated gastric emptying in rats and humans, due to the inhibitory effects of CCK being reduced or diminished due to prolonged exposure to lipids [42, 44]. Özdemir-Kumral and their team used rats and found that a HFD caused inflammation in the gut, disrupted enteric innervations (which resulted in increased gastric contractility), and abolished the inhibitory effects of nesfatin-1 on gastric emptying [42]. However, this and other studies also suggested that the accelerated motility observed with a HFD might be dependent on several factors, such as the: (1) consistency of the food consumed, (2) duration of feeding, (3) method of feeding or fat exposure, and (4) type of fat being consumed, whether it was saturated or unsaturated fat or trans-fat [42]. In our HFD model, whether there are adaptations to or shifts in fat digestion in the larvae, remains to be elucidated.

In the SDG model, the presence of glucose appeared to affect the contractions such that the time for one contraction (in both directions) to start and finish was extended in the anterior and mid intestinal regions, and the interval between contractions was extended in the anterograde (in the MI and PI) and retrograde direction (in the AI and MI). These data indicate that the GI tract exhibited relatively lower motility when larvae were immersed in glucose. The data from our SDG model support those from several other studies, where a prolonged exposure to glucose (or hyperglycaemia) led to changes in hormones that coordinate GI motility [7]. However, we found that in the SDG model, there was a significantly faster anterograde velocity in the mid-intestine when compared with the retrograde velocity. This suggests that like in the SD controls, forward propagation is still dominant in the mid-intestine of the SDG larvae. Thus, even though the overall GI activity is significantly reduced, forward peristalsis is still taking place.

Differences in the expression of genes related to ICC, Nos, the insulin-like growth factor, and appetite and feeding were also investigated in the ED, HFD and SDG models compared to the SD controls. Regarding the ICC-related genes *kitlb* and *CD44* were upregulated in the ED model, and *CD34*, *CD44* and *ANO1* were upregulated in the HFD model, compared with the SD controls. This might indicate an increase in the number of ICC in the ED and HFD larvae. However, the gene expression experiments were conducted with whole larvae and it is known that ICC reside in other regions of the body, not just the intestine [45]. Therefore, the changes in gene expression observed, might not directly indicate that there were more ICC in the gut. In addition, *CD44* is not exclusively expressed in ICC, it is also expressed in mast cells, and it is known to play an important role in cell development. Thus, as the 7 dpf larvae are still developing, the elevated expression of *CD44* might be due to development rather than alterations in gut motility [46]. Anoctamin-1 (encoded by *ANO1*) is a Ca^2+^-activated chloride channel (and a selective marker of ICC), which is crucial for the conductance of slow waves in ICC to modify pacemaker and slow wave activity [47, 48]. Hence, the upregulation of *ANO1* observed in the HFD larvae, suggests that there was increased ICC activity; this might help account for the accelerated GI motility observed in this model.

With regards to the expression of the various *nos*-related genes, *nos1a*, *nos2a* and *nos2b* were all upregulated in the ED model and *nos1a* was upregulated in the SDG model, suggesting that more NO was being produced. NO is known to modulate GI motility in zebrafish as it does in mammals. It is an inhibitory mediator, which leads to smooth muscle relaxation and so this might account for the observed GI actions in the anterior and mid intestinal regions [22]. However, the genetic expression of *nos* is not exclusively in the digestive system as it can be found in (and modulate) the nervous, endocrine and reproductive systems as well. Therefore, repeating these experiments using the dissected GI tract of adult zebrafish (which model the ED and SDG larvae) is required to fully understand the expression levels and effects on GI motility.

Genes related to the insulin-like growth factor were relatively unchanged in the ED, HFD and SDG models compared with the SD controls, except that *igfr1a* was significantly downregulated in the SDG model. This suggests that insulin-like growth factor activity was reduced in this model, which might indicate the presence of insulin resistance [27]. Indeed, a previous study, using gastric smooth muscle cells from rats, found that insulin-like growth factor inhibited gastric cell apoptosis when incubated in a high concentration of glucose [49]. Hence, the downregulation of *igfr1a* observed, might be due to apoptosis of the smooth muscle cells, which resulted in reduced GI motility.

With regards to genes involved in appetite and feeding, there was an upregulation of *ghrelin* expression in the ED and SDG models. In mammals, ghrelin is known to induce appetite and it has prokinetic effects on the GI tract [25]. However, the effects of ghrelin in zebrafish have yet to be elucidated although studies in another teleost, the rainbow trout (*Oncorhynchus mykiss*), indicate that ghrelin exhibits anorexigenic effects and decreases food consumption [25]. Therefore, further investigations are required to explain the upregulation of *ghrelin* observed in zebrafish and how this might regulate the GI effects observed. Certainly, these findings may be supportive of the use of ghrelin agonists in patients with diabetic gastroparesis, and the potential translational value of our studies [50].

The increased expression of *orexin* in the HFD model supports the observation of enhanced motility, as orexin is a hormone that increases food intake and delays the onset of fullness [25]. An increase in the expression of *NYP* was also found in the SDG model. NPY initiates food intake with a preference for carbohydrate and increases feeding [25]. Thus, the upregulated *npy* levels observed in the SDG model might indicate that the larvae were actively feeding and consuming glucose even though deteriorated GI motility was measured. However, further studies to investigate the effects of upregulated *ghrelin* and *NPY* are required to fully understand the observed results.

## Conclusions

We validated three zebrafish larvae models that exhibited diabetes-like phenotypes, by measuring the BMI and adipose deposits, and glucose and insulin assays. Simultaneously, we established a GI motility analysis protocol using 7 dpf larvae in a fasting state after being fed a standardized diet, and we described the main regional differences in motility that were associated with their known functions. Thus, our data reflected the fact that the anterior intestine consists largely of mixing actions, whereas the mid-intestine mainly transports food, and the posterior end is involved in the expulsion of waste, where rectal waves can be observed and quantified [22]. Similar motility patterns were observed in our different DM models, but they differed in the extent of their actions and suggest that diets have significant impact on GI motility. For example, our results indicated that excessive feeding slows down the mixing action in the anterior intestine, whereas a high fat diet initiates progressive motility action, and exposure to glucose results in deteriorated GI motility. Though gastric emptying was not the focus of this study, peristaltic results from the glucose and high-fat diet models could be reflective of the constipation or diarrhoea observed in DM patients [51, 52]. It is likely that the nutritional route to DM could influence the mode of GI dysfunction leading to sub-sets of patients that may require specific approaches to treatment. However, it is crucial to keep in mind that the anterograde and retrograde contractions had to be tracked manually and were therefore prone to experimental bias. Hence, in future studies, it would be beneficial to carry out blind analyses to eliminate such bias. In addition, the genetic expression studies were carried out using whole fish and serve as the limitation of using larvae. To improve upon this, developing adult fish models and repeating the genetic studies using GI tract exclusively is highly recommended [53]. Ultimately, the three zebrafish models characterised in this study will help improve our understanding of GI complications in DM and aid in the development of personalised therapeutics to alleviate GI symptoms using novel anti-diabetic treatments.

## Supporting information

S1 Video S1 Video GI motility and kymograph in 7dpf larvae.avi. (AVI)

S1 Data (XLSX)
